# Ascertainment of Aboriginal and Torres Strait Islander status for assessment of perinatal health outcomes: Reported versus derived maternal ethnicity in Western Australian pregnancy data

**DOI:** 10.1111/ajo.13832

**Published:** 2024-06-04

**Authors:** Ye'elah E. Berman, John P. Newnham, Sarah V. Ward, Kiarna Brown, Dorota A. Doherty

**Affiliations:** ^1^ Division of Obstetrics and Gynaecology, Medical School The University of Western Australia Perth Western Australia Australia; ^2^ Royal Darwin Hospital Darwin Northern Territory Australia; ^3^ Menzies School of Health Research Darwin Northern Territory Australia

**Keywords:** Aboriginal health, identification, data linkage, indigenous, perinatal

## Abstract

**Background:**

Under‐identification of Aboriginal and Torres Strait Islander (hereafter referred to as Aboriginal) people can result in inaccurate estimation of health outcomes. Data linkage has improved identification of Aboriginal people in administrative datasets.

**Aim:**

To compare three methods of ascertainment of Aboriginal status using only pregnancy data from the Western Australian Midwives Notification System (MNS), to the linked Indigenous Status Flag (ISF) derived by the Department of Health.

**Materials and Methods:**

This retrospective population‐based cohort study utilised logistic regression to determine which demographic characteristics were associated with under‐identification, and the effect of ascertainment method on perinatal adverse outcomes.

**Results:**

All methods identified a core group of 19 017 (83.0%) Aboriginal women and the ISF identified 2298 (10.0%) women who were not identified using any other method. Under‐ascertainment was lowest when a woman's Aboriginal status was determined by ever being recorded as Aboriginal in the MNS data, and highest when taken as it had been recorded for the birth in question. Maternal age <20 years, smoking during pregnancy, pre‐existing diabetes, a history of singleton preterm birth and being in the lowest 20% of Socio‐Economic Indexes for Areas score were all associated with a higher chance of being identified by the methods using only the MNS. These methods were less likely to identify nulliparous women, and those with maternal age ≥35 years. The method of ascertainment of Aboriginality did not make a significant difference to the adjusted predicted marginal probabilities of adverse perinatal outcomes.

**Conclusion:**

Unlinked pregnancy data can be used for epidemiological research in Aboriginal obstetric populations.

## INTRODUCTION

The ability to capture accurate data about the health of Aboriginal and Torres Strait Islander people (henceforth referred to as Aboriginal) is crucial to policy development, service delivery, and evaluation of health programs. Administrative datasets can be used for these purposes, and provide information about a large number of individuals over time, without incurring the additional costs of traditional data collection.^1^ While identification of Aboriginal status has improved over time,^2^ Aboriginal people are still under‐identified in administrative datasets.^3^, ^4^, ^5^, ^6^, ^7^ This under‐identification is not uniform across hospitals^8^, ^9^ and can result in inaccurate estimation of health^10^ and mortality^11^ outcomes.

Linkage of the births data to the Registry of Births Deaths and Marriages improved ascertainment of Aboriginal status of birthing mothers in New South Wales by 47%^5^ and babies in Victoria by 87%.^12^ The use of algorithms incorporating Aboriginal status information from many linked datasets and records, over an individual's lifetime and across generations,^13^ has reduced missing data and increased consistency within an individual.^1^ The Western Australian (WA) Data Linkage unit uses a validated algorithm to obtain the derived Aboriginal and Torres Strait Islander Status Flag^14^ (ISF) which to date has been the best method of ascertainment of Aboriginal status in WA linked data. However, the cost or time required to receive linked data for research purposes can be prohibitive, necessitating the use of unlinked or internally linked data.

This study therefore aimed to compare three methods of ascertainment of maternal Aboriginal status using a single, unlinked data set only: the Midwives Notification System (MNS). We determined which method best approximated the linked ISF, which maternal characteristics were associated with a better chance of identification using the MNS‐only algorithms, and what effect using different methods of ascertainment had on estimated rates of key perinatal outcomes.

## MATERIALS AND METHODS

### Study population and data sources

This retrospective population‐based cohort study used data from all WA births between 2009 and 2019 reported in the MNS. The MNS is a registry of births recorded by the attending midwife, including all live and stillbirths of at least 20 weeks gestation, or greater than 400 g birthweight if gestation is unknown. The linked ISF dataset provided the best indication of maternal Aboriginal status, and the Registry of Births Deaths and Marriages (mortality data) was used to determine neonatal deaths. The mortality dataset and the ISF were probabilistically linked to the MNS by Data Linkage Services WA.^15^, ^16^

### Outcomes

The primary outcome was whether or not a woman was identified as Aboriginal by each method of ascertainment of Aboriginal status. Perinatal outcomes of interest were preterm birth (PTB: birth at less than 37 weeks gestation), stillbirth, neonatal death, requirement for serious resuscitation (continuous positive airway pressure, endotracheal intubation, intermittent positive pressure ventilation and external cardiac massage and ventilation) and admission to special care. All perinatal outcomes were derived from the MNS except neonatal death which was defined as death within 28 days of birth and identified using the mortality data.

### Exposure

The primary exposure was method of identification of Aboriginal status, with the linked ISF considered the optimal method for determining the mother's Aboriginal status. To derive the ISF, a multi‐stage median^1^ algorithm is applied to the Aboriginal and/or Torres Strait Islander status variables from the WA Department of Health MNS, Birth Registration, Hospital Morbidity Data Collection, Emergency Department Data Collection and Death Registration datasets,^14^ and an individual is assigned an Aboriginal status of ‘Yes’, ‘No’ or ‘Missing’.^14^ Aboriginal status was also derived for this study using the ethnicity variable in the MNS‐only, in three additional ways.

1. Traditional Unlinked – A woman's Aboriginal status was based on what had been recorded for each of her instances of giving birth.
2. Weight‐of‐Evidence – If it was recorded that a woman was Aboriginal on ≥50% of her births, then she was assigned the status of Aboriginal on all of her instances of giving birth.
3. Ever‐Aboriginal – If it was recorded that a woman was Aboriginal on any of her births, then she was assigned the status of Aboriginal on all of her instances of giving birth.

The Traditional Unlinked method is the only method available for completely unlinked data. If the dataset is internally linked longitudinally (a person is identified as the same person if they appear multiple times within the dataset), then the Weight‐of‐Evidence or Ever‐Aboriginal methods can also be used.

### Potential covariates

Maternal age, parity, smoking during pregnancy, pre‐existing diabetes and hypertension, asthma, multiple pregnancy, gestational diabetes, pre‐eclampsia, caesarean at the last delivery and other maternal conditions were all derived from the MNS and were considered for inclusion in regression models. The other maternal conditions category included any pre‐existing medical conditions diagnosed before the pregnancy, that may influence the pregnancy or pregnancy care.^17^ History of singleton PTB and history of stillbirth were derived for each mother and delivery hospital was used to derive hospital type (established tertiary, evolving tertiary, non‐tertiary). Socio‐economic status was assigned based on the MNS postcode variable for maternal residence, using the Index of Relative Socio‐Economic Advantage and Disadvantage (SEIFA) score, derived by the Australian Bureau of Statistics.^18^ SEIFA scores were categorised into those in the lowest 20%, and those in the upper 80% of scores. Birth method (vaginal birth, instrumental vaginal birth, planned caesarean or emergency caesarean) and gestational age at birth were included when neonatal death, major resuscitation, or special care nursery admission were being modelled.

### Statistical analyses

The number of births to women identified as Aboriginal, by method of ascertainment, as well as under‐ascertainment compared to the ISF were reported, by year. For each of the MNS‐only methods, the number and percent of births in common with the ISF, births identified by the ISF but not by the MNS methods, and births identified by the MNS methods but not by the ISF, were reported. Frequency distributions of demographic characteristics were reported for women identified as Aboriginal by the ISF, and rates of pregnancy and neonatal adverse outcomes were reported by method of identification.

Logistic regression models were used to determine which maternal characteristics were associated with a higher chance of under‐ascertainment by each MNS‐only method, compared to being identified by that method or by the ISF. Logistic regression models that included all women identified as Aboriginal by any method were used to calculate adjusted and unadjusted marginal predicted probabilities of key perinatal outcomes, by method of ascertainment. In these models, a marginal standardisation method^19^ was used to calculate predicted probabilities of PTB, stillbirth, neonatal death, requirement for serious resuscitation and admission to special care. Women with missing SEIFA (*N* = 87, 0.4%) were excluded from all logistic regression models due to small numbers preventing modelling, and pregnancies with multiples (*N* = 644, 2.8%) and terminations of pregnancies (*N* = 27, 0.1%) were excluded from models predicting perinatal adverse events to avoid confounding of outcomes associated with multiple births. Adjusted models included covariates that were statistically significant at the *P* < 0.20 level.

SAS (version 9.4: SAS Institute Inc., Cary, NC, USA) and Stata (StataCorp. 2021. Stata Statistical Software: Release 17. StataCorp LLC., College Station, TX, USA) statistical software packages were used for data analyses. The study was approved by the Women and Newborn Health Service Human Research Ethics Committee (RGS0000002677), the Health Department of Western Australia Human Research Ethics Committee (RGS0000000704) and the WA Aboriginal Health Ethics Committee (965).

## RESULTS

**Figure 1:**
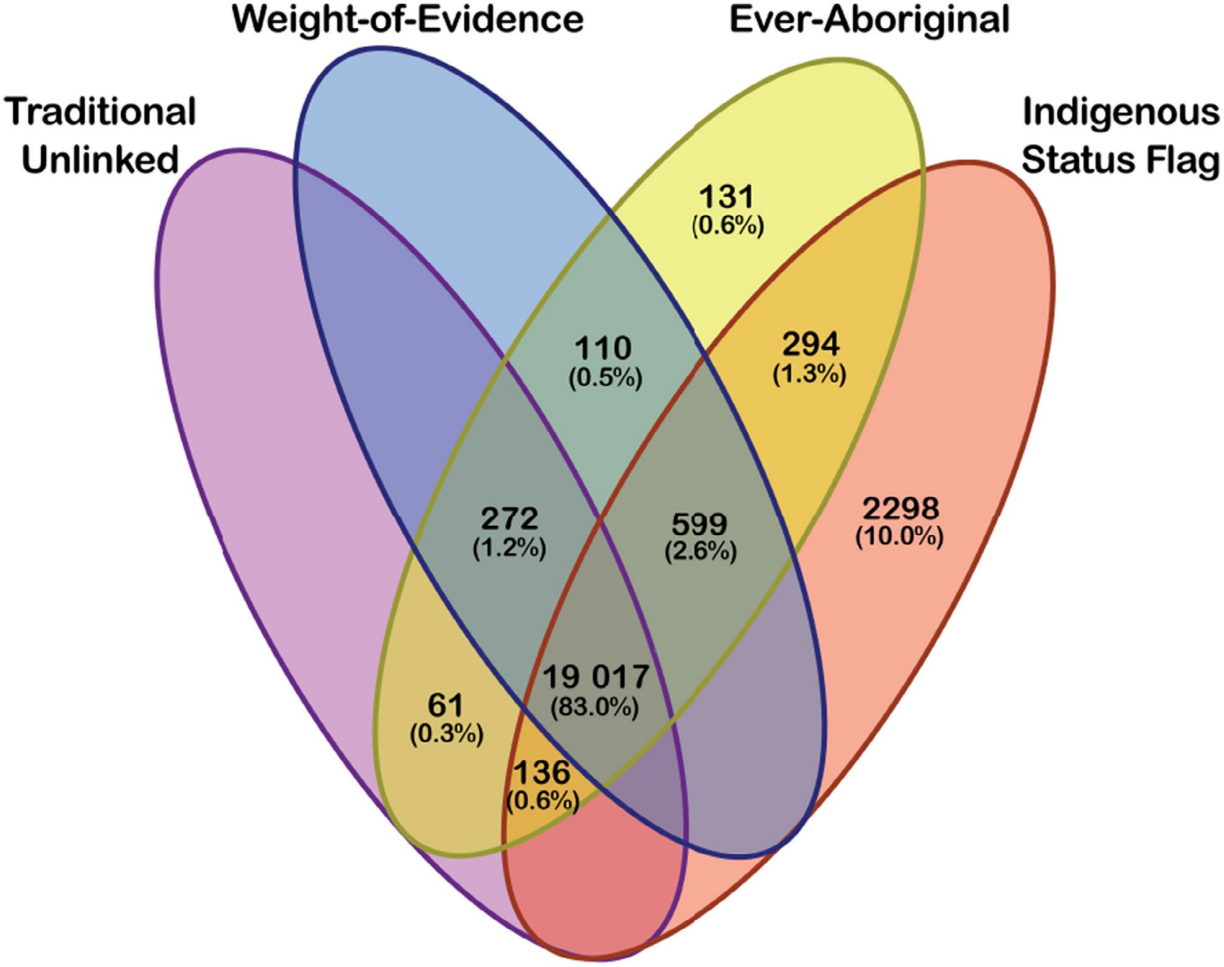
Number of mothers identified by each method of ascertainment of Aboriginal status, and those uniquely identified by each method, for all births to Aboriginal women in Western Australia (2009–2019). ^†^Indigenous Status Flag method (ISF): a multi‐stage median algorithm is applied to the Aboriginal and/or Torres Strait Islander status variables from multiple Western Australia Department of Health datasets to determine Aboriginal status. ^‡^Traditional Unlinked method: a woman's Aboriginal status was based on what had been recorded for each of her instances of giving birth in the Midwives Notification System (MNS). ^§^Weight‐of‐Evidence method: if it was recorded that a woman was Aboriginal on ≥50% of her births in the MNS, then she was assigned the status of Aboriginal on all of her instances of giving birth. ^¶^Ever‐Aboriginal method: if it was recorded that a woman was Aboriginal on any of her births in the MNS, then she was assigned the status of Aboriginal on all of her instances of giving birth.

The four methods of ascertaining Aboriginal status (the ISF and the three MNS‐only methods) identified a combined total of 22 918 Aboriginal women who gave birth in WA between 2009 and 2019. The ISF alone identified 22 344 (97.5%) of these women. All methods identified a core group of 19 017 (83.0%) Aboriginal women. The ISF identified 2298 (10.0%) women as Aboriginal who were not identified using any other method, and 3191 (14.1%) women who were not identified using the Traditional Unlinked method (Fig. 1). The percentage of birthing women in the state who were Aboriginal was at a maximum in 2009 (2079 births, 6.7%) reducing to 5.9% (2005 births) in 2012 and increasing to 6.4% (2136 births) in 2019 (Table 1).

**Table 1:** Number of births to mothers identified as Aboriginal, by year and method of ascertainment (Indigenous Status Flag, Traditional Unlinked, Weight‐of‐Evidence, and Ever‐Aboriginal methods), Western Australia (2009–2019)

Year	Total births in the state	Method of ascertainment for identifying Aboriginality								
		Any method		Indigenous Status Flag^†^	Traditional Unlinked^‡^		Weight‐of‐Evidence^§^		Ever‐Aboriginal^¶^	
	*N*	*n*	% Aboriginal	*n*	*n*	% under‐ascertainment	*n*	% under‐ascertainment	*n*	% under‐ascertainment
2009	31 206	2079	6.7	2003	1763	12.0	1814	9.4	1880	6.1
2010	31 256	2016	6.4	1962	1700	13.4	1754	10.6	1806	8.0
2011	32 195	2042	6.3	1966	1740	11.5	1791	8.9	1851	5.8
2012	33 862	2005	5.9	1963	1656	15.6	1731	11.8	1798	8.4
2013	34 400	2096	6.1	2031	1757	13.5	1816	10.6	1876	7.6
2014	35 206	2127	6.0	2075	1803	13.1	1839	11.4	1898	8.5
2015	34 981	2083	6.0	2032	1743	14.2	1802	11.3	1866	8.2
2016	35 890	2151	6.0	2116	1834	13.3	1890	10.7	1941	8.3
2017	34 546	2109	6.1	2074	1803	13.1	1839	11.3	1893	8.7
2018	33 426	2074	6.2	2037	1820	10.7	1833	10.0	1873	8.1
2019	33 304	2136	6.4	2085	1867	10.5	1889	9.4	1938	7.1

^†^ Indigenous Status Flag method: A multi‐stage median algorithm is applied to the Aboriginal and/or Torres Strait Islander status variables from multiple Western Australia Department of Health datasets to determine Aboriginal status.

^‡^ Traditional Unlinked method: A woman's Aboriginal status was based on what had been recorded for each of her instances of giving birth in the Midwives Notification System (MNS).

^§^ Weight‐of‐Evidence method: If it was recorded that a woman was Aboriginal on ≥50% of her births in the MNS, then she was assigned the status of Aboriginal on all of her instances of giving birth.

^¶^ Ever‐Aboriginal method: If it was recorded that a woman was Aboriginal on any of her births in the MNS, then she was assigned the status of Aboriginal on all of her instances of giving birth.

A higher % under‐ascertainment indicates that fewer women who were identified by the Indigenous Status Flag, were identified by the MNS‐only method.

The patterns of agreement between the ISF and the MNS ascertainment methods are shown in Table S1. Under‐ascertainment overall was 10.0, 12.0 and 14.1% using the Ever‐Aboriginal, Weight‐of‐Evidence and Traditional Unlinked methods, respectively. Under‐ascertainment was higher (more women who were identified by the ISF were not identified by the MNS‐only methods) at the tertiary hospital (Traditional Unlinked = 15.1%, Weight‐of‐Evidence = 13.3%, Ever‐Aboriginal = 11.7%) than at non‐tertiary hospitals (Traditional Unlinked = 14.7%, Weight‐of‐Evidence = 12.4%, Ever‐Aboriginal = 10.1%).

Table 2 shows unadjusted and adjusted associations between maternal characteristics, and under‐identification by the MNS‐only methods. Women who were identified as Aboriginal by the ISF but not by the MNS‐only methods, were less likely to be younger than 20 years of age, smoke during pregnancy, be in the lowest 20% of SEIFA scores, have pre‐existing diabetes or have had a previous PTB. Women who were identified as Aboriginal by the ISF but not by the MNS‐only methods, were more likely to be nulliparous, have asthma, have had a caesarean at their last delivery, and be greater than or equal to 35 years of age. There was a significant association between parity and identification using the MNS‐only methods with nulliparous women comprising 30.4% of women identified by the ISF, and 35.3% of the women identified by the ISF but not by the Traditional Unlinked method (adjusted odds ratio (aOR): 1.5, 95% CI: 1.3, 1.6). Consequently, this analysis was repeated, stratified by parity (nulliparous vs multiparous). Results showed the directions of association were similar between these two groups of women (Tables S2 and S3).

**Table 2:** Frequencies and percentages of maternal characteristics by ascertainment method, and associations between maternal characteristics and under‐ascertainment in the MNS‐only methods of Aboriginal identification, compared to the ISF, in Western Australia (2009–2019)

Maternal demographics	Indigenous Status Flag^†^ (*N* = 22 260)	In the Indigenous Status Flag, not in Traditional Unlinked^‡^ (*N* = 3179)			In the Indigenous Status Flag, not in Weight‐of‐Evidence^††^ (*N* = 2717)			In the Indigenous Status Flag, not in Ever‐Aboriginal^¶^ (*N* = 2287)		
	*N*	*N*	Unadjusted OR (95% CI)	Adjusted OR (95% CI)	*N*	Unadjusted OR (95% CI)	Adjusted OR (95% CI)	*N*	Unadjusted OR (95% CI)	Adjusted OR (95% CI)
Maternal age										
<20	3863 (17.4)	**421 (13.2)**	**0.7 (0.6–0.8)**	**0.5 (0.5–0.6)**	**346 (12.7)**	**0.7 (0.6–0.8)**	**0.5 (0.5–0.6)**	**290 (12.7)**	**0.7 (0.6–0.8)**	**0.5 (0.4–0.6)**
20–34	16 499 (74.1)	2472 (77.8)	ref	ref	2118 (78.0)	ref	ref	**1757 (76.8)**	**ref**	**ref**
≥35	1898 (8.5)	286 (9.0)	1.0 (0.9–1.1)	1.1 (1.0–1.3)	253 (9.3)	1.0 (0.9–1.2)	**1.2 (1.0–1.4)**	**240 (10.5)**	**1.2 (1.0–1.4)**	**1.4 (1.2–1.6)**
Nulliparous	6770 (30.4)	**1122 (35.3)**	**1.3 (1.2–1.4)**	**1.5 (1.3–1.6)**	**945 (34.8)**	**1.3 (1.2–1.4)**	**1.5 (1.3–1.6)**	**869 (38.0)**	**1.5 (1.3–1.6)**	**1.8 (1.6–2.0)**
Smoked during pregnancy	10 001 (44.9)	**1047 (32.9)**	**0.6 (0.5–0.6)**	**0.6 (0.6–0.7)**	**894 (32.9)**	**0.6 (0.5–0.6)**	**0.6 (0.6–0.7)**	**745 (32.6)**	**0.6 (0.5–0.6)**	**0.6 (0.6–0.7)**
SEIFA in the lowest 20%	6935 (31.2)	**576 (18.1)**	**0.4 (0.4–0.5)**	**0.5 (0.4–0.5)**	**486 (17.9)**	**0.4 (0.4–0.5)**	**0.5 (0.4–0.5)**	**407 (17.8)**	**0.5 (0.4–0.5)**	**0.5 (0.4–0.6)**
Maternal conditions										
Pre‐existing diabetes	531 (2.4)	**41 (1.3)**	**0.5 (0.4–0.7)**	**0.5 (0.4–0.7)**	**38 (1.4)**	**0.6 (0.4–0.8)**	**0.6 (0.4–0.8)**	**35 (1.5)**	**0.6 (0.4–0.9)**	**0.6 (0.4–0.9)**
Pre‐existing hypertension	285 (1.3)	43 (1.4)	1.0 (0.8–1.5)		34 (1.3)	1.0 (0.7–1.4)		33 (1.4)	1.1 (0.8–1.6)	
Asthma	2190 (9.8)	**470 (14.8)**	**1.7 (1.6–1.9)**	**1.6 (1.5–1.8)**	**402 (14.8)**	**1.7 (1.5–1.9)**	**1.6 (1.4–1.8)**	**351 (15.3)**	**1.8 (1.6–2.0)**	**1.7 (1.5–1.9)**
Other maternal conditions	9288 (41.7)	1291 (40.6)	1.0 (0.9–1.0)	0.9 (0.9–1.0)	1131 (41.6)	1.0 (0.9–1.1)		968 (42.3)	1.0 (0.9–1.1)	
Multiple pregnancy	626 (2.8)	98 (3.1)	1.1 (0.9–1.3)		96 (3.5)	1.3 (1.0–1.6)	1.3 (1.0–1.6)	70 (3.1)	1.1 (0.9–1.4)	
Gestational diabetes	1713 (7.7)	252 (7.9)	1.0 (0.9–1.2)		205 (7.5)	1.0 (0.8–1.1)	0.9 (0.7–1.0)	185 (8.1)	1.1 (0.9–1.3)	
Pre‐eclampsia	687 (3.1)	94 (3.0)	0.9 (0.8–1.2)		77 (2.8)	0.9 (0.7–1.1)	0.8 (0.7–1.1)	72 (3.1)	1.0 (0.8–1.3)	
Obstetric history										
Previous singleton PTB										
No	9699 (43.6)	1395 (43.9)	ref	ref	1202 (44.2)	ref	ref	938 (41.0)	ref	ref
Yes	3249 (14.6)	**317 (10.0)**	**0.7 (0.6–0.7)**	**0.7 (0.6–0.8)**	**281 (10.3)**	**0.7 (0.6–0.8)**	**0.7 (0.6–0.9)**	**215 (9.4)**	**0.7 (0.6–0.8)**	**0.7 (0.6–0.8)**
Unknown	2542 (11.4)	345 (10.9)	0.9 (0.8–1.1)	0.9 (0.8–1.1)	289 (10.6)	0.9 (0.8–1.0)	0.9 (0.8–1.1)	265 (11.6)	1.1 (0.9–1.3)	1.0 (0.9–1.2)
Previous stillbirth	627 (2.8)	**55 (1.7)**	**0.6 (0.4–0.8)**	0.8 (0.6–1.0)	48 (1.8)	0.6 (0.4–0.8)	0.8 (0.6–1.1)	38 (1.7)	0.6 (0.4–0.8)	0.8 (0.6–1.1)
Caesarean last delivery	3171 (14.2)	479 (15.1)	1.1 (1.0–1.2)	**1.2 (1.1–1.4)**	**426 (15.7)**	**1.1 (1.0–1.3)**	**1.3 (1.1–1.4)**	**346 (15.1)**	**1.1 (1.0–1.2)**	**1.3 (1.1–1.4)**

^**†**^ Indigenous Status Flag (ISF): A multi‐stage median algorithm is applied to the Aboriginal and/or Torres Strait Islander status variables from multiple WA Department of Health datasets to determine Aboriginal status.

^**‡**^ Traditional Unlinked method: A woman's Aboriginal status was based on what had been recorded for each of her instances of giving birth in the Midwives Notification System (MNS).

^§^ Weight‐of‐Evidence method: If it was recorded that a woman was Aboriginal on ≥50% of her births in the MNS, then she was assigned the status of Aboriginal on all of her instances of giving birth.

^¶^ Ever‐Aboriginal method: If it was recorded that a woman was Aboriginal on any of her births in the MNS, then she was assigned the status of Aboriginal on all of her instances of giving birth.

87 births were excluded as they had missing SEIFA status and therefore could not be included in regression modelling.

Adjusted logistic regression models include covariates that were significant at the *P* < 0.20 level.

Bolded text indicates that the maternal characteristic is significantly associated with identification with the MNS‐only method versus the ISF at the *P* < 0.05 level.

OR, odds ratio; PTB, preterm birth; SEIFA, Index of Relative Socio‐Economic Advantage and Disadvantage.

The method of ascertainment of Aboriginality did not make a statistically significant difference to the adjusted predicted marginal probabilities of any of the adverse perinatal outcomes including PTB, stillbirth, neonatal death major resuscitation and special care nursery admission (Table 3). However, at the individual level it did make a large difference to the cell counts, with the ISF identifying 1140 more special care nursery admissions than the Traditional Unlinked method, and 893 more than the Weight‐of‐Evidence method.

**Table 3:** Unadjusted and adjusted marginal predicted probabilities of adverse perinatal outcomes, by method of ascertainment of Aboriginal status, for Aboriginal women who gave birth to singletons in Western Australia between 2009 and 2019

	*n*	Unadjusted predicted probability	Adjusted predicted probability
		Marginal mean (95% CI)	Marginal mean (95% CI)
Preterm birth			
Indigenous Status Flag^†^	3006	13.9 (13.4–14.4)	13.9 (13.4–14.3)
Traditional Unlinked^‡^	2686	14.3 (13.8–14.7)	14.0 (13.5–14.5)
Weight‐of‐Evidence^§^	2731	14.1 (13.6–14.6)	13.9 (13.5–14.4)
Ever‐Aboriginal^¶^	2795	14.0 (13.5–14.5)	13.9 (13.4–14.3)
Stillbirth			
Indigenous Status Flag	254	1.2 (1.0–1.3)	1.2 (1–1.3)
Traditional Unlinked	226	1.2 (1.0–1.4)	1.2 (1.0–1.3)
Weight‐of‐Evidence	227	1.2 (1.0–1.3)	1.2 (1.0–1.3)
Ever‐Aboriginal	230	1.2 (1.0–1.3)	1.2 (1.0–1.3)
Neonatal death			
Indigenous Status Flag	86	0.4 (0.3–0.5)	0.4 (0.3–0.5)
Traditional Unlinked	82	0.4 (0.3–0.5)	0.4 (0.3–0.5)
Weight‐of‐Evidence	81	0.4 (0.3–0.5)	0.4 (0.3–0.5)
Ever‐Aboriginal	83	0.4 (0.3–0.5)	0.4 (0.3–0.5)
Major Resuscitation^*^			
Indigenous Status Flag	2848	13.3 (12.9–13.8)	13.3 (12.9–13.7)
Traditional Unlinked	2498	13.4 (12.9–13.9)	13.3 (12.9–13.8)
Weight‐of‐Evidence	2562	13.4 (12.9–13.9)	13.3 (12.9–13.8)
Ever‐Aboriginal	2606	13.2 (12.7–13.7)	13.2 (12.8–13.7)
Admission to special care nursery			
Indigenous Status Flag	7646	35.8 (35.2–36.4)	35.8 (35.2–36.4)
Traditional Unlinked	6506	34.9 (34.3–35.6)	34.9 (34.2–35.5)
Weight‐of‐Evidence	6753	35.3 (34.6–36.0)	35.2 (34.6–35.9)
Ever‐Aboriginal	7010	35.5 (34.9–36.2)	35.5 (34.9–36.2)

^*^ Major Resuscitation refers only to continuous positive airway pressure, endotracheal intubation, intermittent positive pressure ventilation and external cardiac massage and ventilation.

^†^ Indigenous Status Flag method (ISF): A multi‐stage median algorithm is applied to the Aboriginal and/or Torres Strait Islander status variables from multiple Western Australia Department of Health datasets to determine Aboriginal status.

^‡^ Traditional Unlinked method: A woman's Aboriginal status was based on what had been recorded for each of her instances of giving birth in the Midwives Notification System (MNS).

^§^ Weight‐of‐Evidence method: If it was recorded that a woman was Aboriginal on ≥50% of her births in the MNS, then she was assigned the status of Aboriginal on all of her instances of giving birth.

^¶^ Ever‐Aboriginal method: If it was recorded that a woman was Aboriginal on any of her births in the MNS, then she was assigned the status of Aboriginal on all of her instances of giving birth.

670 births were excluded as they were multiple births or terminations of pregnancy. Terminations of pregnancy were identified as inductions resulting in intrapartum death at the established tertiary centre before 22 weeks or resulting in antepartum death between 22 and 24 weeks.

An additional 85 births were excluded as they had missing SEIFA status and therefore could not be included in regression modelling.

Stillbirths were removed from calculations in which neonatal death, resuscitation of admission to special care nursery were the outcome.

Adjusted regression models include covariates that were significant at the *P* < 0.20 level.

PTB, preterm birth; SEIFA, Index of Relative Socio‐Economic Advantage and Disadvantage.

## DISCUSSION

Our study examined the agreement in assignment of maternal Aboriginal status between the ISF, obtained using linked data, and three methods based only on the MNS data. We also quantified the effect that the differences had on estimation of rates of adverse perinatal outcomes, and determined which maternal characteristics were associated with under‐identification using the MNS‐only methods. All three methods identified 83.0% of Aboriginal women at the state level; however, 10.0% of Aboriginal births identified by the ISF were not identified by any of the MNS‐only methods. Rates of adverse outcomes when using the ISF were comparable to that of the three other ascertainment methods.

The Ever‐Aboriginal method had the lowest under‐ascertainment of Aboriginal women giving birth in WA. This was the least likely method to ‘miss’ Aboriginal women, but also the most likely to assign Aboriginal status to women not identified as Aboriginal by the ISF. In the absence of the ISF, the optimal method of ascertainment of Aboriginal status would depend on the project being conducted: whether it was most important that Aboriginal women were not missed (Ever‐Aboriginal method), or that non‐Aboriginal women were not included in the sample (Traditional Unlinked method). The Weight‐of‐Evidence method provides a balanced approach to these ascertainment issues. Our results were reasonably consistent with other Australian data, with a NSW study showing that a multi‐stage median algorithm enhanced identification by 11.4% compared to the Traditional Unlinked method.^20^ By comparison, our ISF enhanced identification by 14.7% over the Traditional Unlinked method (although our algorithm included death data and theirs did not).

Logistic regression models predicting whether a woman identified as Aboriginal in the ISF but was not identified as Aboriginal by the MNS‐only methods, showed that maternal age <20 years, smoking during pregnancy, the lowest 20% of SEIFA, pre‐existing diabetes and a history of singleton PTB were all associated with lower under‐ascertainment using the MNS‐only methods. These characteristics are all likely to be associated with additional contact with the healthcare team during pregnancy, and therefore potentially result in more accurate recording of the woman's Aboriginal status. It is surprising that women who had a caesarean at their last delivery were less likely to be identified as Aboriginal. Considering these women had given birth before, they would likely have had another birth recorded in the MNS (unless their first birth occurred before the start of our study period). Having more than one pregnancy in the MNS should increase the likelihood of being identified as Aboriginal using the Weight‐of‐Evidence and Ever‐Aboriginal methods as even if the woman was not recorded as Aboriginal on the birth in question, she may have been assigned Aboriginal status based on how she reported her ethnicity in a previous pregnancy. This finding persisted, even when the models were only run on multiparous women. The ISF identified an additional 10.0% of women as Aboriginal who were not identified by any MNS‐only method, indicating that these women had been identified as Aboriginal in other linked datasets, or for births outside of our study period. Considering 83% of women were identified by all four methods, it is not surprising that the unadjusted and adjusted predicted probabilities of adverse perinatal outcomes did not significantly differ by method of ascertainment. However, this lack of difference in the odds ratios does indicate that the rates of adverse outcomes among the women identified by the ISF, and the women missed, were similar. This is an important finding as it means that where linked data is not available to researchers, unlinked or internally linked data can be used for calculation of rates of adverse neonatal outcomes.

Finally, Aboriginality is self‐reported in the relevant datasets, and it must be acknowledged that there are complex reasons as to why a woman may choose to identify as Aboriginal on one occasion, but not on another. Furthermore, for a woman to identify as Aboriginal, the question must actually be asked of her by the health provider. The National best practice guidelines for collecting Indigenous status^21^ were released in 2010 to create a systematic national approach to the collection, recording and reporting of Aboriginal status in health datasets. Subsequently, National best practice guidelines for data linkage activities relating to Aboriginal and Torres Strait Islander people^22^ were developed to support analysts and data custodians to use Indigenous status in linked data appropriately while being guided by the core values of Aboriginal and Torres Strait Islander human research.

Our study utilised a population‐based cohort study design which minimised systematic error. We also had access to 11 years of data for the whole of WA, which provided a large study population. It is known that remoteness of residence is related to severe neonatal morbidity and mortality,^23^ as well as PTB and low birthweight.^24^ While remoteness is correlated to SEIFA score, our inability to include remoteness in our modelling could be limiting our ability to appropriately adjust for all maternal characteristics, in turn affecting our conclusions.

In conclusion, for the period 2009–2019, we determined that using the Traditional Unlinked method is adequate for identification of Aboriginal women in the MNS. Where the MNS is internally linked, use of the Weight‐of‐Evidence or Ever‐Aboriginal methods provides a marginal improvement. The ISF remains the optimal way for identifying Aboriginal women in linked data, but the other three methods result in similar rates of adverse perinatal outcomes. In summary, the stand‐alone MNS dataset can be used (with caution) for epidemiological research in Aboriginal obstetric populations.

## CONFLICT OF INTEREST STATEMENT

The authors declare they have no competing interests.

## DATA ACCESSIBILITY STATEMENT

The authors do not have permission to share patient‐level data extracted from the Data Linkage Unit of the Department of Health of WA. Data can only be made available to researchers who apply to the Department of Health of WA's Human Research Ethics Committee (https://ww2.health.wa.gov.au/Articles/A_E/Department‐of‐Health‐Human‐Research‐Ethics‐Committee) and Data Linkage Unit (www. datalinkagewa.org.au). Please contact the lead author, Ye'elah Berman (yeelah.berman@uwa.edu.au) to further discuss the availability of data.

## Supporting information


**Table S1.** Ascertainment of maternal Aboriginal status using the Traditional Unlinked, Weight‐of‐Evidence, and Ever‐Aboriginal methods, compared to the Indigenous Status Flag (ISF), for the state and by hospital type (2009–2019).
**Table S2.** Frequencies and percentages of maternal characteristics by ascertainment method, and associations between maternal characteristics and under‐ascertainment in the Western Australian Midwives Notification System (MNS) only methods of Aboriginal identification, compared to the Indigenous Status Flag (ISF), nulliparous women in Western Australia (2009–2019).
**Table S3.** Frequencies and percentages of maternal characteristics by ascertainment method, and associations between maternal characteristics and under‐ascertainment in the Western Australian Midwives Notification System (MNS) only methods of Aboriginal identification, compared to the Indigenous Status Flag (ISF), multiparous women in Western Australia (2009–2019).
