# Supplementary material for: Ascertainment of Aboriginal and Torres Strait Islander status for assessment of perinatal health outcomes: Reported versus derived maternal ethnicity in Western Australian pregnancy data
Source: Aust N Z J Obstet Gynaecol. 2024 Jun 4;64(6):556–65. doi: 10.1111/ajo.13832 (PMC11683754; doi:10.1111/ajo.13832)
Supplement: Supplementary file 1 — Table S1. Ascertainment of maternal Aboriginal status using the Traditional Unlinked, Weight‐of‐Evidence, and Ever‐Aboriginal methods, compared to the Indigenous Status Flag (ISF), for the state and by hospital type (2009–2019). Table S2. Frequencies and percentages of maternal characteristics by ascertainment method, and associations between maternal characteristics and under‐ascertainment in the Western Australian Midwives Notification System (MNS) only methods of Aboriginal identification, compared to the Indigenous Status Flag (ISF), nulliparous women in Western Australia (2009–2019). Table S3. Frequencies and percentages of maternal characteristics by ascertainment method, and associations between maternal characteristics and under‐ascertainment in the Western Australian Midwives Notification System (MNS) only methods of Aboriginal identification, compared to the Indigenous Status Flag (ISF), multiparous women in Western Australia (2009–2019). [file AJO-64-556-s001.docx]

**Supplementary Table 1. Ascertainment of maternal Aboriginal status using the Traditional Unlinked, Weight-of-Evidence, and Ever-Aboriginal methods, compared to the Indigenous Status Flag (ISF), for the state and by hospital type (2009-2019)**

| **State-wide** | **Traditional Unlinked**^‡^ **(N=22,677)** | | **Weight-of-Evidence**^§^ **(N=22,726)** | | **Ever-Aboriginal**^¶^ **(N=22,918)** | |
| --- | --- | --- | --- | --- | --- | --- |
| (22,344 births estimated by the ISF^†^, including 628 multiple births) | **N** | **%** | **N** | **%** | **N** | **%** |
| Births in common with the ISF | 19153 | 84.5 | 19616 | 86.3 | 20046 | 87.5 |
| In the ISF but not in the other cohort | 3191 | 14.1 | 2728 | 12.0 | 2298 | 10.0 |
| In the other cohort but not in the ISF | 333 | 1.5 | 382 | 1.7 | 574 | 2.5 |
| **Tertiary hospital** | **Traditional Unlinked**^‡^ **(N=5,638)** | | **Weight-of-Evidence**^§^ **(N=5,644)** | | **Ever-Aboriginal**^¶^ **(N=5,685)** | |
| (5,587 births estimated by the ISF^†^, including 386 multiple births) | **N** | **%** | **N** | **%** | **N** | **%** |
| Births in common with the ISF | 4734 | 84.0 | 4837 | 85.7 | 4924 | 86.6 |
| In the ISF but not in the other cohort | 853 | 15.1 | 750 | 13.3 | 663 | 11.7 |
| In the other cohort but not in the ISF | 51 | 0.9 | 57 | 1.0 | 98 | 1.7 |
| **Non-Tertiary hospitals** | **Traditional Unlinked**^‡^ **(N=14,141)** | | **Weight-of-Evidence**^§^ **(N=14,178)** | | **Ever-Aboriginal**^¶^ **(N=14,310)** | |
| (13,897 births estimated by the ISF^†^, including 214 multiple births) | **N** | **%** | **N** | **%** | **N** | **%** |
| Births in common with the ISF | 11813 | 83.5 | 12140 | 85.6 | 12450 | 87.0 |
| In the ISF but not in the other cohort | 2084 | 14.7 | 1757 | 12.4 | 1447 | 10.1 |
| In the other cohort but not in the ISF | 244 | 1.7 | 281 | 2.0 | 413 | 2.9 |

Abbreviations: PTB: Preterm birth

^†^Indigenous Status Flag method (ISF): A multi-stage median algorithm is applied to the Aboriginal and/or Torres Strait Islander status variables from multiple Western Australia Department of Health datasets to determine Aboriginal status.

^‡^Traditional Unlinked method: A woman’s Aboriginal status was based on what had been recorded for each of her instances of giving birth in the Midwives Notification System (MNS).

^§^Weight-of-Evidence method: If it was recorded that a woman was Aboriginal on ≥50% of her births in the MNS, then she was assigned the status of Aboriginal on all of her instances of giving birth.

^¶^Ever-Aboriginal method: If it was recorded that a woman was Aboriginal on any of her births in the MNS, then she was assigned the status of Aboriginal on all of her instances of giving birth.

^††^A second tertiary centre commenced in 2015. As it is still evolving, it has a much lower PTB rate than the established tertiary centre, but a higher PTB rate than the non-tertiary centres. As a result, it is included in the state-wide analysis, but not in the sub-analyses by hospital type.

**Supplementary Table 2. Frequencies and percentages of maternal characteristics by ascertainment method, and associations between maternal characteristics and under ascertainment in the MNS only methods of Aboriginal identification, compared to the ISF, nulliparous women in Western Australia (2009-2019)**

|  | **Indigenous Status Flag^†^ (N=6,770)** | **In the Indigenous Status Flag, not in Traditional Unlinked^‡^ (N=1,122)** | | | **In the Indigenous Status Flag, not in Weight-of-Evidence**^§^ **(N=945)** | | | **In the Indigenous Status Flag, not in Ever-Aboriginal**^¶^ **(N=869)** | | |
| --- | --- | --- | --- | --- | --- | --- | --- | --- | --- | --- |
| **Maternal Demographics** | **N** | **N** | **Unadjusted OR (95% CI)** | **Adjusted OR (95% CI)** | **N** | **Unadjusted OR (95% CI)** | **Adjusted OR (95% CI)** | **N** | **Unadjusted OR (95% CI)** | **Adjusted OR (95% CI)** |
| Maternal age |  |  |  |  |  |  |  |  |  |  |
| <20 | 3026 (44.7) | **336 (29.9)** | **0.5 (0.4, 0.6)** | **0.5 (0.4, 0.6)** | **277 (29.3)** | **0.5 (0.4, 0.6)** | **0.5 (0.4, 0.6)** | **242 (27.8)** | **0.5 (0.4, 0.5)** | **0.5 (0.4, 0.6)** |
| 20-34 | 3604 (53.2) | 746 (66.5) |  |  | **633 (67)** |  |  | **592 (68.1)** |  |  |
| ≥35 | 140 (2.1) | **40 (3.6)** | **1.5 (1, 2.1)** | 1.3 (0.9, 2) | **35 (3.7)** | **1.5 (1, 2.2)** | 1.4 (0.9, 2.1) | **35 (4)** | **1.6 (1.1, 2.4)** | 1.5 (1, 2.2) |
| Smoked during pregnancy | 2618 (38.7) | **293 (26.1)** | **0.5 (0.4, 0.6)** | **0.5 (0.5, 0.6)** | **244 (25.8)** | **0.5 (0.4, 0.6)** | **0.5 (0.5, 0.6)** | **226 (26)** | **0.5 (0.4, 0.6)** | **0.6 (0.5, 0.7)** |
| SEIFA in the lowest 20% | 2030 (30) | **162 (14.4)** | **0.3 (0.3, 0.4)** | **0.4 (0.3, 0.5)** | **140 (14.8)** | **0.4 (0.3, 0.4)** | **0.4 (0.3, 0.5)** | **130 (15)** | **0.4 (0.3, 0.5)** | **0.4 (0.4, 0.5)** |
| **Maternal Conditions** |  |  |  |  |  |  |  |  |  |  |
| Pre-existing diabetes | 98 (1.4) | 12 (1.1) | 0.7 (0.4, 1.3) | 0.6 (0.3, 1.2) | 12 (1.3) | 0.9 (0.5, 1.6) |  | 12 (1.4) | 1 (0.5, 1.8) |  |
| Pre-existing hypertension | 71 (1) | 13 (1.2) | 1.1 (0.6, 2) |  | 8 (0.8) | 0.8 (0.4, 1.6) | 0.6 (0.3, 1.2) | 8 (0.9) | 0.9 (0.4, 1.8) | 0.6 (0.3, 1.3) |
| Asthma | **696 (10.3)** | **169 (15.1)** | **1.7 (1.4, 2.1)** | **1.6 (1.3, 2)** | **139 (14.7)** | **1.6 (1.3, 2)** | **1.5 (1.2, 1.9)** | **128 (14.7)** | **1.6 (1.3, 2)** | **1.5 (1.2, 1.9)** |
| Other maternal conditions | 2808 (41.5) | **432 (38.5)** | **0.9 (0.8, 1)** | **0.8 (0.7, 1)** | 378 (40) | 0.9 (0.8, 1.1) |  | 356 (41) | 1 (0.8, 1.1) |  |
| Multiple pregnancy | 137 (2) | **36 (3.2)** | **1.8 (1.2, 2.7)** | 1.4 (0.9, 2.1) | **34 (3.6)** | **2.1 (1.4, 3.1)** | **1.6 (1.1, 2.5)** | **32 (3.7)** | **2.1 (1.4, 3.2)** | **1.6 (1.1, 2.5)** |
| Gestational diabetes | 422 (6.2) | 71 (6.3) | 1 (0.8, 1.3) | 1.2 (0.9, 1.6) | 57 (6) | 1 (0.7, 1.3) | 1.3 (1, 1.7) | 56 (6.4) | 1.1 (0.8, 1.4) |  |
| Pre-eclampsia | 356 (5.3) | 59 (5.3) | 1 (0.7, 1.3) |  | 46 (4.9) | 0.9 (0.7, 1.3) |  | 46 (5.3) | 1 (0.7, 1.4) |  |

Abbreviations: SEIFA: Index of Relative Socio-Economic Advantage and Disadvantage

**^†^**Indigenous Status Flag: A multi-stage median algorithm is applied to the Aboriginal and/or Torres Strait Islander status variables from multiple WA Department of Health datasets to determine Aboriginal status.

**^‡^** Traditional Unlinked method: A woman’s Aboriginal status was based on what had been recorded for each of her instances of giving birth in the Midwives Notification System (MNS).

^§^Weight-of-Evidence method: If it was recorded that a woman was Aboriginal on ≥50% of her births in the MNS, then she was assigned the status of Aboriginal on all of her instances of giving birth.

^¶^Ever-Aboriginal method: If it was recorded that a woman was Aboriginal on any of her births in the MNS, then she was assigned the status of Aboriginal on all of her instances of giving birth

**^††^**21 births were excluded as they had missing SEIFA status and therefore could not be included in regression modelling.

**^‡‡^**Adjusted logistic regression models include covariates that were significant at the p<.20 level.

**^§§^**Bolded text indicates that the maternal characteristic is significantly associated with identification with the MNS-only method versus the ISF at the p<.05 level. **Supplementary Table 3. Frequencies and percentages of maternal characteristics by ascertainment method, and associations between maternal characteristics and under ascertainment in the MNS only methods of Aboriginal identification, compared to the ISF, multiparous women in Western Australia (2009-2019)**

|  | **Indigenous Status Flag  (N=15,490)** | **In the Indigenous Status Flag, not in Traditional Unlinked (N=2,057)** | | | **In the Indigenous Status Flag, not in Weight-of-Evidence (N=1,772)** | | | **In the Indigenous Status Flag, not in Ever-Aboriginal (N=1,418)** | | |
| --- | --- | --- | --- | --- | --- | --- | --- | --- | --- | --- |
| **Maternal Demographics** | **N** | **N** | **Unadjusted OR (95% CI)** | **Adjusted OR (95% CI)** | **N** | **Unadjusted OR (95% CI)** | **Adjusted OR (95% CI)** | **N** | **Unadjusted OR (95% CI)** | **Adjusted OR (95% CI)** |
| Maternal age |  |  |  |  |  |  |  |  |  |  |
| <20 | 837 (5.4) | **85 (4.1)** | **0.7 (0.6, 0.9)** | **0.7 (0.6, 0.9)** | **69 (3.9)** | **0.7 (0.5, 0.9)** | **0.7 (0.5, 0.9)** | **48 (3.4)** | **0.6 (0.5, 0.8)** | **0.6 (0.5, 0.8)** |
| 20-34 | 12895 (83.2) | 1726 (83.9) |  |  | 1485 (83.8) |  |  | 1165 (82.2) |  |  |
| ≥35 | 1758 (11.3) | 246 (12) | 1 (0.9, 1.2) | 1.1 (0.9, 1.3) | 218 (12.3) | 1.1 (0.9, 1.2) | 1.1 (1, 1.3) | **205 (14.5)** | **1.3 (1.1, 1.5)** | **1.3 (1.1, 1.6)** |
| Smoked during pregnancy | 7383 (47.7) | **754 (36.7)** | **0.6 (0.5, 0.7)** | **0.6 (0.6, 0.7)** | **650 (36.7)** | **0.6 (0.6, 0.7)** | **0.7 (0.6, 0.7)** | **519 (36.6)** | **0.6 (0.6, 0.7)** | **0.7 (0.6, 0.7)** |
| SEIFA in the lowest 20% | 4905 (31.7) | **414 (20.1)** | **0.5 (0.5, 0.6)** | **0.5 (0.5, 0.6)** | **346 (19.5)** | **0.5 (0.4, 0.6)** | **0.5 (0.5, 0.6)** | **277 (19.5)** | **0.5 (0.4, 0.6)** | **0.5 (0.5, 0.6)** |
| **Maternal Conditions** | **N** |  | **Unadjusted OR (95% CI)** | **Adjusted OR (95% CI)** |  | **Unadjusted OR (95% CI)** | **Adjusted OR (95% CI)** |  | **Unadjusted OR (95% CI)** | **Adjusted OR (95% CI)** |
| Pre-existing diabetes | 433 (2.8) | **29 (1.4)** | **0.5 (0.3, 0.7)** | **0.5 (0.3, 0.7)** | **26 (1.5)** | **0.5 (0.3, 0.7)** | **0.5 (0.3, 0.8)** | **23 (1.6)** | **0.6 (0.4, 0.9)** | **0.5 (0.3, 0.8)** |
| Pre-existing hypertension | 214 (1.4) | 30 (1.5) | 1 (0.7, 1.5) |  | 26 (1.5) | 1.1 (0.7, 1.6) |  | 25 (1.8) | 1.3 (0.9, 2) | 1.3 (0.9, 2.1) |
| Asthma | 1494 (9.6) | **301 (14.6)** | **1.8 (1.5, 2)** | **1.7 (1.4, 1.9)** | **263 (14.8)** | **1.8 (1.5, 2)** | **1.7 (1.4, 1.9)** | **223 (15.7)** | **1.9 (1.6, 2.2)** | **1.8 (1.5, 2)** |
| Other maternal conditions | 6480 (41.8) | 859 (41.8) | 1 (0.9, 1.1) |  | 753 (42.5) | 1 (0.9, 1.1) |  | 612 (43.2) | 1.1 (1, 1.2) |  |
| Multiple pregnancy | 489 (3.2) | 62 (3) | 0.9 (0.7, 1.2) |  | 62 (3.5) | 1.1 (0.9, 1.5) |  | 38 (2.7) | 0.8 (0.6, 1.2) |  |
| Gestational diabetes | 1291 (8.3) | 181 (8.8) | 1.1 (0.9, 1.3) |  | 148 (8.4) | 1 (0.8, 1.2) |  | 129 (9.1) | 1.1 (0.9, 1.4) |  |
| Pre-eclampsia | 331 (2.1) | 35 (1.7) | 0.8 (0.5, 1.1) | 0.8 (0.6, 1.1) | 31 (1.7) | 0.8 (0.5, 1.1) |  | 26 (1.8) | 0.8 (0.6, 1.3) |  |
| **Obstetric History** | **N** |  | **Unadjusted OR (95% CI)** | **Adjusted OR (95% CI)** |  | **Unadjusted OR (95% CI)** | **Adjusted OR (95% CI)** |  | **Unadjusted OR (95% CI)** | **Adjusted OR (95% CI)** |
| Previous singleton PTB |  |  |  |  |  |  |  |  |  |  |
| No | 9699 (62.6) | 1395 (67.8) |  |  | 1202 (67.8) |  |  | 938 (66.1) |  |  |
| Yes | 3249 (21) | **317 (15.4)** | **0.7 (0.6, 0.7)** | **0.7 (0.6, 0.8)** | **281 (15.9)** | **0.7 (0.6, 0.8)** | **0.7 (0.6, 0.9)** | **215 (15.2)** | **0.7 (0.6, 0.8)** | **0.7 (0.6, 0.8)** |
| Unknown | 2542 (16.4) | 345 (16.8) | 0.9 (0.8, 1.1) | 1 (0.8, 1.1) | 289 (16.3) | 0.9 (0.8, 1) | 0.9 (0.8, 1.1) | 265 (18.7) | 1.1 (0.9, 1.3) | 1.1 (0.9, 1.2) |
| Previous stillbirth | 627 (4) | **55 (2.7)** | **0.6 (0.5, 0.8)** | 0.8 (0.6, 1) | **48 (2.7)** | **0.6 (0.5, 0.9)** | 0.8 (0.6, 1.1) | **38 (2.7)** | **0.6 (0.5, 0.9)** | 0.8 (0.6, 1.1) |
| Caesarean last delivery | 3171 (20.5) | **479 (23.3)** | **1.2 (1.1, 1.4)** | **1.2 (1.1, 1.4)** | **426 (24)** | **1.3 (1.1, 1.4)** | **1.3 (1.1, 1.4)** | **346 (24.4)** | **1.3 (1.1, 1.5)** | **1.3 (1.1, 1.4)** |

Abbreviations: PTB: Preterm birth; SEIFA: Index of Relative Socio-Economic Advantage and Disadvantage

**^†^**Indigenous Status Flag: A multi-stage median algorithm is applied to the Aboriginal and/or Torres Strait Islander status variables from multiple WA Department of Health datasets to determine Aboriginal status.

**^‡^** Traditional Unlinked method: A woman’s Aboriginal status was based on what had been recorded for each of her instances of giving birth in the Midwives Notification System (MNS).

^§^Weight-of-Evidence method: If it was recorded that a woman was Aboriginal on ≥50% of her births in the MNS, then she was assigned the status of Aboriginal on all of her instances of giving birth.

^¶^Ever-Aboriginal method: If it was recorded that a woman was Aboriginal on any of her births in the MNS, then she was assigned the status of Aboriginal on all of her instances of giving birth

**^††^**66 births were excluded as they had missing SEIFA status and therefore could not be included in regression modelling.

**^‡‡^**Adjusted logistic regression models include covariates that were significant at the p<.20 level.

**^§§^**Bolded text indicates that the maternal characteristic is significantly associated with identification with the MNS-only method versus the ISF at the p<.05 level.
